# Comparative Shear-Bond Strength of Six Dental Self-Adhesive Resin Cements to Zirconia

**DOI:** 10.3390/ma8063306

**Published:** 2015-06-05

**Authors:** Si-Eun Lee, Ji-Hyeon Bae, Jae-Won Choi, Yong-Chan Jeon, Chang-Mo Jeong, Mi-Jung Yoon, Jung-Bo Huh

**Affiliations:** Department of Prosthodontics, Dental Research Institute, Biomedical Research Institute, School of Dentistry, Pusan National University, Yangsan, Gyeongnam 626-770, Korea; E-Mails: oopooh@hanmail.net (S.-E.L.); bjhyeon@gmail.com (J.-H.B.); won9180@hanmail.net (J.-W.C.); jeonyc@paran.com (Y.-C.J.); cmjeong@pusan.ac.kr (C.-M.J.); p-venus79@hanmail.net (M.-J.Y.)

**Keywords:** self-adhesive cements, zirconia, shear bond strength, no pretreatment

## Abstract

This study compared shear bond strength (SBS) of six self-adhesive resin cements (SARC) and one resin-modified glass ionomer cement (RMGIC) to zirconia before and after thermocycling. The cylinder shape (Φ 2.35 mm × 3 mm) of six SARCs (G-CEM LinkAce (GLA), Maxcem Elite (MAX), Clearfil SA Luting (CSL), PermaCem 2.0 (PM2), Rely-X U200 (RXU), Smartcem 2 (SC2)) were bonded to the top surface of the zirconia specimens with light-curing. RMGIC (Fujicem (FJC)) was bonded to the specimens with self-curing. The shear bond strength of all cemented specimens was measured with universal testing machine. Half of the specimens were thermocycled 5000 times before shear bonding strength testing. Fractured surfaces were examined with a field-emission SEM (10,000×) and analyzed by energy dispersive x-ray analysis. MAX, PM2, SC2 group without thermocycling and GLA, MAX, PM2 group with thermocycling showed adhesive failure, but GLA, CSL, RXU, FJC group without thermocycling and SLC, RXU, SC2, FJC group with thermocycling indicated cohesive failure. Within the limitation of this study, All of SARCs except MAX demonstrated higher bond strength than that of RMGIC regardless of thermocycling. Also, SARC containing MDP monomers (CSL) retained better bonds than other cements.

## 1. Introduction

Adequate adhesion between restorations and teeth is one of the major factors of successful indirect restoration [1]. For adequate adhesion, cements suitable to the restoration type must be selected [2]. An ideal luting cement must provide sustainable bonds with different materials, sufficient compression and tensile strengths, wettability, and resistance to dissolution in the oral cavity [3,4]. There are various types of cement: zinc phosphate cement (ZPC), poly carboxylate cement, glass ionomer cement, resin-modified glass ionomer cement (RMGIC), and resin cement [5]. Resin cement has better compressive and tensile strengths, toughness, resilience, and extremely lower solubility than other luting agents. Moreover, it is aesthetically excellent and provides several color option [6].

In conventional resin cements, a pretreatment procedure is required to achieve adhesion, and the procedure is complicated. When the moisture-proofing or dentin wettability is not properly maintained, the bond strengths could weaken [7]. A self-etching system was developed to simplify the treatment procedure and to prevent the collapse of the collagen fiber in the dentin through acid etching. However, the permeation of moisture through the adhesives can cause the bond strength to deteriorate when hardening is delayed [8]. To overcome this problem, self-adhesive resin cement (SARC) that combines the adhesives and cements were developed. These cements offer the mechanical, aesthetic, and adhesive advantages of typical resin cements [9], and they also do not require pretreatments, owing to their acidic functional monomer [10].

Demands for all-ceramic restorations have been increasing, as aesthetic dental treatments are increasingly required [11]. Especially, zirconia is widely used based on the CAD/CAM technology, which improves the process and precision level [12,13]. Since zirconia has a high strength [14], it can be cemented using a typical ZPC or RMGIC [15]. However, when resin cements are used, the retentive force to restorations can be enhanced [16,17], marginal sealing can be improved, and the fracture strength of restorations can increase [18].

To increase bond strengths between resin cements and zirconia, studies on various surface treatments have been conducted [1,19,20,21]. Acid-etching on zirconia surface have been attempted to enhance micromechanical retentivity, but hydrofloric acid etching was unsuccessful because zirconia is a polycrystalline structure [2]. Sandblasting was used to enhance the mechanical bond strengths, and was reported to have contributed to the improvement of the bond strength [22]. In other studies, however, the pretreatment degraded the bond strength [16,23,24].

Another method of enhancing bond strengths is silane treatment, which can improve chemical bonding. The silane-coupling agents do not work because zirconia does not contain silica [21,25]. Some studies reported that the bond strengths increased after the tribochemical silica/silane-coating [26,27]; but based on other studies, the strength decreased after thermocycling [25,28].

In the meantime, efforts to use SARCs for bonding zirconia are on the rise. Even though SARC does not require any pretreatments to zirconia surface, many clinicians usually perform pretreatments on zirconia surface prior to use of SARC like as using conventional resin cement. Also, the strengths of SARCs to untreated zirconia surface have been rarely evaluated.

The purpose of this study was to investigate the shear bond strength of self-adhesive resin cements to zirconia without surface pretreatment and its failure.

## 2. Results and Discussion

### 2.1. Results

#### 2.1.1. Shear Bond Strength Comparison According to the Cement Type

The means and standard deviations of the shear bond strengths of the SARCs and the control group are shown in Table 1. Before thermocycling, the shear bond strength in Group PM2, GLA, SC2, and CSL showed greater value than those in Group MAX, RXU, and FJC. After thermocycling, the greatest bonding strength was manifested in Group CSL. All SARCs, except MAX, showed greater shear bond strength than that of RMGIC cement regardless of whether or not to thermal cycling. The mean shear bond strengths of most of the resin cements decreased after thermocycling. Reduction in bond strength was greater with group GLA (32.8%), followed by Groups PM2 (28.6%) and MAX (27.3%). In case of groups RXU and SC2, however, they did not show a statistically significant difference after thermocycling (*p* > 0.05), also Group CSL showed a significant increase (*p* < 0.05) (Table 1).

**Table 1:** Mean shear bond strength values (MPa), standard deviations(SDs), and independent *t*-test *p*-values of the cements on the zirconia surface before and after the thermocycling.

Group	n	Before			After		Independent t-test *p*-value
		Mean	*SD*		Mean	*SD*	
G-CEM LinkAce	10	3.96^a^	*0.56*		2.66^c,d^	*0.53*	0.000
Maxcem Elite	10	2.86^b^	*0.61*		2.08^d^	*0.46*	0.004
Clearfill SA Luting	10	3.90^a^	*0.58*		4.62^a^	*0.60*	0.014
PermaCem 2.0	10	4.19^a^	*0.66*		2.99^b,c^	*0.57*	0.000
RelyX U200	10	2.84^b^	*0.61*		2.36^c,d^	*0.41*	0.056
SmartCem 2	10	3.93^a^	*0.48*		3.44^b^	*0.59*	0.056
FujiCEM	10	1.74^c^	*0.72*		2.23^d^	*0.42*	0.154

Note: In the same column, superscripts that differ in each cell indicate a statistical difference (*p* < 0.05), and the same superscript letters indicate cements that showed insignificant differences under the same testing conditions via *post hoc* analysis using the Tukey test.

#### 2.1.2. Scanning Electron Microscope Observation and Surface Analysis

**Figure 1:**
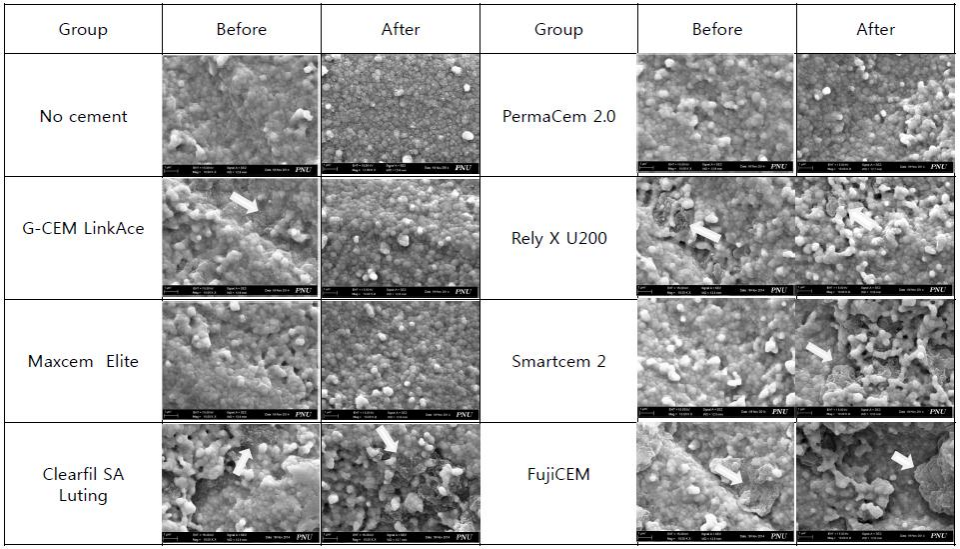
Field-emission scanning microscope images of the fractured zirconia surface before and after thermocycling. The white arrows represent the cements on the zirconia surface and those means that they showed cohesive failure. (magnification: 10,000×).

The fracture surfaces were examined through a scanning electron microscope, and the results of the component analyses through EDAX are shown in Figure 1. The arrow in the diagram indicates the remaining resin cement, which was confirmed by analyzing its major component, silica (Si). In groups MAX, PM2, and SC2, adhesive failure showed before thermocycling; and in groups GLA, CSL, and RXU, cohesive failure was noted. After thermocycling, adhesive failure manifested in groups GLA, MAX, and PM2, and cohesive failure was observed in groups CSL, RXU, and SC2. In the group FJC, cohesive failure was exhibited regardless of the thermocycling.

### 2.2. Discussion

This study compared the shear bond strengths of the self-adhesive cements to zirconia without surface pretreatment between thermocycling and non-thermocycling. The shear bond strengths of six SARCs bonded with untreated zirconia surface were significantly greater than that of FJC prior to the thermocycling. These results corresponded with those of previous studies that confirmed the greater bond strength of resin cement than that of RMGIC [16,29].

The main component of SARCs is phosphate functional monomer. When phosphate monomer is applied to zirconia, the hydrogen group of phosphate monomer and the oxygen group of zirconia slowly react to produce water molecules and to form a stable Zr-O-P covalent bond [10,30]. There must be sufficient wettability in the early stage of the reaction to use the hydrophilic property, but excessive hydrophilicity can cause swelling that can adversely affect dimensional stability and mechanical strength, so the hydrophobicity must increase after the initial reaction. The phosphoric acid monomers for dental usage include MHP (6-methacryloxyhexyl dihydrogen phosphate), GPDM (glycerol phosphate dimethacrylate), BisHEMA-Phosphate (2-methacryloxyethyl dihydrogen phosphate), PENTA (dipentaerythritol pentaacrylate monophosphate), and MDP (10-methacryloxydecyl dihydrogen phosphate) [10,31,32].

In this study, group PM2 showed the greatest shear bond strength before thermocycling, but the differences among Groups PM2, GLA, SC2, and CSL were insignificant. According to the manufacturer, PermaCem 2.0 includes primer components for zirconia in its cement, so no additional primer is required for a high bond strength level. Its low-pH components have self-etching effects that facilitate direct bonding with zirconia. Nevertheless, the manufacturer did not release the details of the cement components, so these are based on estimations [33].

To compare the bond strength according to the storage time, half of the samples were kept in a distilled water bath for 24 h at 37 °C and thermocycling under the following conditions: 5000-cycle at the 5 °C and 55 °C thermal circulation water baths was performed on the others. Thermocycling can simulate the oral cavity environment, and be used as a reference for the artificial aging process [34,35]. Yap *et al.* [36] suggested that the 5000 cycles were clinically equivalent to approximately six months. According to Harper *et al.* [37], a dwell time of 15 s or longer was inappropriate since patients cannot tolerate the direct long-term contact of vital teeth with too cold or hot materials. Accordingly, water bath duration was set at 15 s in this study. As a result, the mean value of shear bond strength in SARCs decreased after 5000 thermal cycling (*p* < 0.05). These outcomes were reported in previous studies [38]. However, the mean bond strengths of Groups RXU and SC2 decreased after thermocycling, but the difference was statistically insignificant (*p* > 0.05). It meant that the shear bond strength of the two cements was maintained with thermocycling. PENTA monomer of SmartCem 2 and phosphoric acid methacrylate of RelyX U200 might be estimated to role as a factor of sustaining bond strength. Group CSL exceptionally showed a statistically significant increase in strength after the thermocycling process (*p* < 0.05). The same result has been reported in previous studies [39]. Clearfil SA Luting contains MDP which is a phosphate monomer with a longer chain of -(CH_2_)_n_ than other phosphoric acid monomers [10,40]. Since this longer backbone could increase the hydrophobicity of the cement, its bond strength was considered greater than that of other self- adhesive resin cements with different phosphate monomers [10]. The result of this study is similar to that of previous ones about the SARC including MDP monomers. In the case of Maxcem Elite, it showed weaker strength than other SARCs due to its GPDM monomer component. GPDM plays the roles of self-etching and bonding [40], and its phosphoric acid molecules are known to etch the enamel and dentin [41]. However, information on the initial-stage acidity, which may be crucial for understanding the chemical properties of bonding to the zirconia surface, is unavailable. Eventually, the acid component of Maxcem becomes insufficient for triggering chemical bonding, and consequently, high bond strength may not be produced.

When the fracture patterns of the samples were seen with the naked eye, adhesive failure was noted in all the samples. However, when the fracture patterns were observed with a field-emission scanning electron microscope and analyzed with EDAX, part of the high-bond-strength cement group showed cohesive failures. Group GLA showed cohesive failure before thermocycling, but adhesive failure after the process, which G-CEM LinkAce was thought to have been affected by aging. Group SC2 showed adhesive failure before thermocycling, but cohesive failure was manifested after thermocycling. This might be attributed to the increased chemical bond of SmartCem 2 due to the thermal circulation. In the case of FujiCEM, cohesive fracture was observed because its own strength was weaker than that of the SARCs relatively, which resulted in fractures in the cement. 

Since this study was conducted to evaluate the specific interaction between each cement type and the zirconia restoration, a simple design for attaching the cement to the zirconia was used. To obtain more realistic results in clinical cases, the bond strength may need to be measured after the teeth and the restoration are bonded, after which the fracture surface may need to be examined. Also, it may be required to calculate in percentage of the bonding area for identifying the type of bonding failure mode with a light microscope. [42] Accordingly, the clinical application must be carefully performed because the bonding mechanism of SARCs has not been fully investigated yet through long-term clinical studies. It is not advisable to determine the bonding ability of new types of cements simply based on the differences in their shear bond strengths. Further comparative studies on additional physical properties such as tensile strength, flexure strength, wettability, and color change may be needed in the future.

## 3. Experimental Section

### 3.1. Tested Materials

In the experiment group, 6 SARCs—G-CEM LinkAce (GLA) (GC Corporation, Tokyo, Japan), Maxcem Elite (MAX) (Kerr, Orange, CA, USA), Clearfil SA Luting (CSL) (Kuraray Medical Co., Osaka, Japan), PermaCem 2.0 (PM2) (DMG, Hamburg, Germany), RelyX U200 (RXU) (3M ESPE, St. Paul, MN, USA), and SmartCem 2 (SM2) (Dentsply Caulk, Yorks, PA, USA)—were used. In the control group, FujiCEM (FJC) (GC Corporation, Tokyo, Japan), which is a RMGIC that does not require pretreatment, was used. The composition of each material is shown in Table 2.

### 3.2. Zirconia Sample Preparation

A total of 140 zirconia samples with an 8.0 mm diameter and a 15.0 mm height were prepared through two-hour sintering at a final temperature of 1500 °C using cylindrical zirconia blocks (Zirtooth, HASS, Kangneung, Korea).

### 3.3. Test Material Bond

The zirconia samples were polished 20 times using 600-grit sandpaper, and were subsequently steam-cleansed and dried. Self-adhesive cements were injected into the samples via the Ultradent plastic hole (Ultradent Product, Inc., South Jordan, UT, USA) [43] to create a cylinder with a 2.35 mm diameter and a 3.0 mm height, and then tack-cured for five seconds using a curing light (Elipar™ FreeLight™ 2, 3M ESPE, St. Paul, MN, USA). After the cylinder was separated from the hole, photopolymerization was additionally performed for 40 s. In the case of FujiCEM, the cement cylinder was separated from the hole after 5 minutes of self-curing. Cured specimens were subsequently kept in a distilled water bath for 24 h at 37 °C [44].

### 3.4. Thermocycling

The specimens were fabricated and then removed from the alignment device, stored in 37 °C distilled water, and then half of them were thermocycled for 5000 cycles between 5 and 55 °C with a dwelling time of 15 s. When not under heat stimulation, the samples were kept in a 37 °C distilled water bath. After thermocycling was completed, the shear bond strength of the samples was measured.

### 3.5. Measurement of the Shear Bond Strength

The bonded specimens were placed in a zig to test their shear bond strength and loaded to failure with a crosshead speed of 1 mm/min using a Universal Testing Machine (Model 3345, Instron Co., Canton, MA, USA). The loads were converted to MPa by dividing the maximum failure load (N) by the bonding area (mm^2^) [44].

**Table 2:** Tested materials, type, and composition.

Material (Lot #)	Manufactures	Type	Composition
G-CEM LinkAce (1309191)	GC (GC Corporation, Tokyo, Japan)	Dual-cure Self adhesive Auto Mix	Paste A: Fluoroalumino silicate glass, Initiator, Urethane dimethacrylate (UDMA), Dimethacrylate, Pigment, Silicon dioxide, Inhibitor Paste B: Silicon dioxide, UDMA, Dimethacrylate, Initiator, Inhibitor
Maxcem Elite (5150785)	Kerr (Kerr, Orange, CA, USA)	Dual-cure Self adhesive	Glycerol phosphate dimethacryalte (GPDM), Co-monomers, Proprietary self-curing redox activator, Camphorquinone, Stabilizer, Barium glass fillers, Fluoroalumino silicate glass filler, silica
Clearfill SA Luting (00388A)	Kuraray (Kuraray Medical Co. Osaka, Japan)	Dual-cure Self adhesive Hand Mix	Paste A: Bisphenol A glycidyl methacrylate(Bis-GMA), Triethyleneglycol dimethacrylate (TEGDMA), 10- methacryloxydecyl dihydrogen phosphate (MDP), Dimethylamine (DMA), Silanated barium glass filler, Silanated colloidal silica Paste B: Bis-GMA, DMA, Silanated barium glass filler, Silanated colloidal silica, surface treated sodium fluoride
PermaCem 2.0 (709149)	DMG(DMG, Hamburg, Germany)	Dual-cure Self adhesive Auto mix	Bis-GMA-based matrix, Barium glass filler content Ethoxylated, TEGDMA, 2-Hydroxyethyl methacrylate (HEMA)
RelyX U200 (551581)	3M ESPE (3M/ESPE, St. Paul, MN, USA)	Dual-cure Self adhesive	Base paste: Methacrylate monomers containing phosphoric acid groups, Methacrylate monomers, Silanated fillers, Initiator components, Stabilizers, Rheological additives Catalyst paste: Methacrylate monomers, Alkaline(basic) fillers, Silanated fillers, Initiator components, Stabilizers, Pigments, Rheological additives
SmartCem 2 (140311)	Dentsply intl (Dentsply Caulk, Yorks, PA, USA)	Dual-cure Self adhesive	UDMA, Urethane modified Bis-GMA, TEGDMA dimethacrylate resins, Dipentaerythritol penttacrylate monophosphate (PENTA), Barium boron fluoroaluminosilicate glass amorphous silica
FujiCEM (1312121)	GC (GC Corporation, Tokyo, Japan)	Self-cure, Resin modified glass ionomer cement	Aluminum silicate glass

### 3.6. Scanning Electron Microscopic Observation and Surface Component Analysis 

The specimen surfaces were examined with a field-emission scanning electron microscope (PRA 40VP, Carl-Zeiss AG, Oberkochen, Germany) at the original magnification of 10,000× to assess the mode of failure and the surface microstructure. The surface of the specimens that were observed using the field-emission scanning electron microscope was analyzed through energy-dispersive x-ray analysis (EDAX) (APOLLO XPP, AMETEK EDAX, Mahwah, NJ, USA).

### 3.7. Statistical Analysis

All the statistical analyses were performed with SPSS version 21.0 (SPSS Inc., Chicago, IL, USA). The normal distribution and homogeneity of variance were checked using the Shapiro-Wilk test and Levene tests, respectively. After the one-way ANOVA test was conducted to compare the shear bond strengths of the different types of cements, the Tukey test was used as a *post hoc* analysis. Through the independent sample t-test, the values before and after thermocycling were compared [44]. A significance level of 0.05 was set for all the tests.

## 4. Conclusions

The comparison of the shear bond strengths of six self-adhesive resin cements, and one resin-modified glass ionomer cement to non-pretreated zirconia before and after 5000 thermocycles led to the following conclusions. Overall, higher bond strengths were demonstrated for most of SARCs compared to RMGIC before thermocycling. However, some SARCs showed similar bonding strength compared to RMGIC after thermocycling. Especially, SARCs containing MDP monomers (CSL) provide continuous and better bonding strength than other cements after thermocycling.
